# Endoscopic closure after endoscopic submucosal dissection using novel hooking attachment with a knotting chain shaped thread system

**DOI:** 10.1055/a-2721-9388

**Published:** 2025-11-05

**Authors:** Hirohito Mori, Yasunori Yamamoto, Kazuki Niida, Masaaki Tange, Yoichi Hiasa

**Affiliations:** 1 Department of Advanced and Innovative Endoscopy, Ehime University Graduate School of Medicine, Toon, Japan; 2 Department of Gastroenterology and Metabology, Ehime University Graduate School of Medicine, Toon, Japan

There were many reports related to closure of artificial ulcer to prevent post-endoscopic submucosal dissection (ESD) bleeding in gastric cancer 1 2 , and a few reports usefulness of endoscopic suturing devices 3 4 . We developed a novel method for post-ESD ulcer closure, hooking attachment with a knotting chain shaped thread system (HAWKS), which requires only conventional sutures and clips. Chain shaped thread with 8-mm small rings was made from 4–0 PDS suture (Johnson & Johnson, Japan). The endoscopic variceal injection sclerotherapy (EIS) balloon (TOP, Japan) was used to store chain suture within a balloon gap ( Fig. 1 **a** ). The balloon was mounted on an endoscope ( Fig. 1 **b** ) 5 . One arm of the Zeoclip (Zeon Medical, Japan) was fixed to the attachment with 4‑0 PDS, completing the HAWKS setup ( Fig. 1 **c** , Video 1 ).

**Fig. 1:**
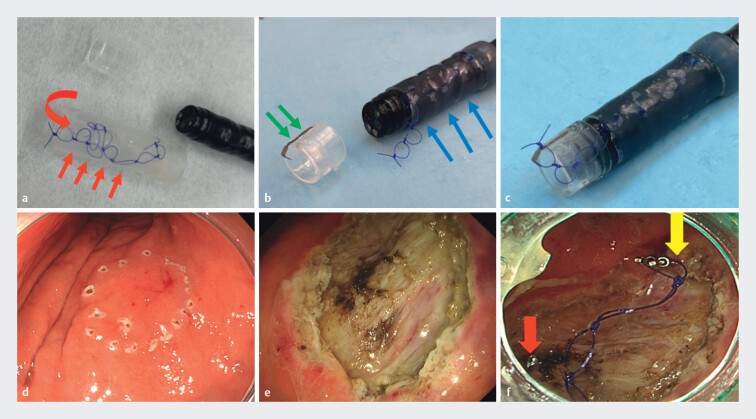
Setup of hooking attachment with the knotting chain shaped thread system and clinical case. **a** Chain shaped thread with 8-mm small rings was made from 4 to 0 PDS monofilament suture. The endoscopic variceal injection sclerotherapy (EIS) balloon (TOP, Japan) was used to store the chain suture within the balloon gap (red arrows). **b** The balloon was mounted on an endoscope (blue arrows). One arm of the Zeoclip was fixed to the attachment with 4–0 PDS (green arrows). **c** Completing of the HAWKS setup. **d, e** A 56-year-old man with 5 cm gastric cancer in greater curvature of upper body underwent ESD. **f** After the chain shaped thread was grasped with the clip, inserted into stomach, and fixed to the anal side of ulcer (yellow arrows). The chain shaped thread was withdrawn from the EIS balloon gap, and the posterior end was clipped to the oral side (red arrows).

Endoscopic closure after endoscopic submucosal dissection using novel hooking attachment with the knotting chain shaped thread system.Video 1

A 56-year-old man with 5 cm gastric cancer in greater curvature of upper body underwent ESD ( Fig. 1 **d, e** ). After chain thread was grasped with clip and fixed to anal side of ulcer, the posterior end was clipped to the oral side withdrawn from the EIS balloon gap ( Fig. 1 **f** ). The anterior portion of the chain thread was clipped to the anterior edge of ulcer, followed by clipping the posterior portion to the posterior edge. After five clip fixations, ulcer floor was approximated ( Fig. 2 **a** ). The pulling ring of the chain thread with 10 o’clock hooking attachment resulted in further inversion and approximation of ulcer floor ( Fig. 2 **b** ). Using the HAWKS-equipped endoscope under traction, the MANTIS clip (Boston Scientific, USA) was deployed through a working channel to achieve complete inverted closure ( Fig. 2 **c, d** ), resulting in no dead space ( Fig. 2 **e** ). Follow-up endoscopy at 1 month confirmed intact closure without any cavity formation ( Fig. 2 **f** ). As we successfully conducted complete closures of four patients using HAWKS in the lesser and greater curvature of gastric body, fornix and antrum, HAWKS enables the complete inverted closure of post-ESD ulcers using only equipment mounted on the endoscope tip with a conventional clip through a working channel.

**Fig. 2:**
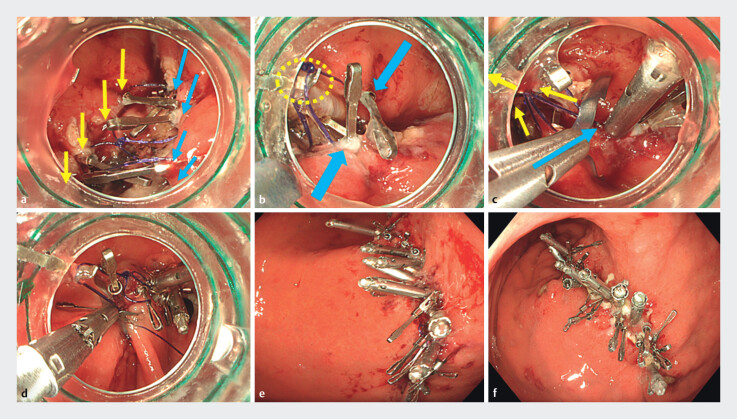
Procedure of the knotting chain shaped thread system in a clinical case and the closure site at 1 month after ESD. **a** Anterior portion of the chain shaped thread was clipped to the anterior edge of ulcer (yellow arrows), followed by clipping the posterior portion to the posterior edge (blue arrows). After five clip fixations, ulcer floor was approximated. **b** Pulling the small ring of the chain shaped thread with hooking attachment (yellow dot circle) resulted in further inversion and approximation of the ulcer floor (blue arrows). **c, d** Using the HAWKS-equipped endoscope under traction (yellow arrows), the MANTIS clip was deployed through the working channel (blue arrow) to achieve complete inverted closure. **e** Complete inverted closure of the ulcer floor, leaving no dead space. **f** Follow-up endoscopy at 1 month demonstrated intact closure without post ESD bleeding and cavity formation.

Endoscopy_UCTN_Code_TTT_1AO_2AO

## Notes

Endoscopy E-Videos https://eref.thieme.de/e-videos
*E-Videos* is an open access online section of the journal *Endoscopy* , reporting on interesting cases and new techniques in gastroenterological endoscopy. All papers include a high-quality video and are published with a Creative Commons CC-BY license. Endoscopy E-Videos qualify for HINARI discounts and waivers and eligibility is automatically checked during the submission process. We grant 100% waivers to articles whose corresponding authors are based in Group A countries and 50% waivers to those who are based in Group B countries as classified by Research4Life (see: https://www.research4life.org/access/eligibility/ ). This section has its own submission website at https://mc.manuscriptcentral.com/e-videos .
